# GPU optimization techniques to accelerate optiGAN—a particle simulation GAN

**DOI:** 10.1088/2632-2153/ad51c9

**Published:** 2024-06-13

**Authors:** Anirudh Srikanth, Carlotta Trigila, Emilie Roncali

**Affiliations:** 1 Department of Biomedical Engineering, University of California, Davis, Davis, CA, United States of America; 2 Department of Radiology, University of California, Davis, Davis, CA, United States of America

**Keywords:** generative adversarial networks, graphics processing unit, performance optimization, radiation detector, multidimensional probability distributions, Monte-Carlo simulation

## Abstract

The demand for specialized hardware to train AI models has increased in tandem with the increase in the model complexity over the recent years. Graphics processing unit (GPU) is one such hardware that is capable of parallelizing operations performed on a large chunk of data. Companies like Nvidia, AMD, and Google have been constantly scaling-up the hardware performance as fast as they can. Nevertheless, there is still a gap between the required processing power and processing capacity of the hardware. To increase the hardware utilization, the software has to be optimized too. In this paper, we present some general GPU optimization techniques we used to efficiently train the optiGAN model, a Generative Adversarial Network that is capable of generating multidimensional probability distributions of optical photons at the photodetector face in radiation detectors, on an 8GB Nvidia Quadro RTX 4000 GPU. We analyze and compare the performances of all the optimizations based on the execution time and the memory consumed using the Nvidia Nsight Systems profiler tool. The optimizations gave approximately a 4.5x increase in the runtime performance when compared to a naive training on the GPU, without compromising the model performance. Finally we discuss optiGANs future work and how we are planning to scale the model on GPUs.

## 1. Introduction

The field of Generative AI has witnessed a significant rise in the utilization of Generative Adversarial Networks (GANs) (Goodfellow *et al* 2014) in recent years. GANs are a class of deep learning networks that are trained to generate realistic data samples. Different types of GAN models are used to produce different data-types such as texts, images, numerical, speeches (Yu *et al* 2017, Isola *et al* 2018, Xu and Veeramachaneni 2018, Donahue *et al* 2019). One such application of a GAN model is optiGAN, a GAN capable of generating complex multidimensional probability distributions (Trigila *et al* 2023). The goal of optiGAN is to mitigate the computational complexity of optical Monte Carlo simulations. Such simulations are extensively used to understand, optimize, and design nuclear medicine systems and high energy physics detectors, but are burdened by the track-wise approach to particle transport of commonly used simulators. OptiGAN demonstrated to be capable of generating multidimensional distributions (energy, position, direction, and time) of optical photons at the photodetector face. This significantly improves optical photon transport modeling in simulations to test new radiation detector technology in large clinical and preclinical imaging systems.

OptiGAN employs a WGAN (Wasserstein GAN) (Arjovsky *et al* 2017) architecture with Gradient Penalty (GP) (Gulrajani *et al* 2017) to generate optical photon distributions trained on accurate optical Monte Carlo simulations generated using GATE (Jan *et al* 2004), a toolkit dedicated to numerical simulations in medical imaging and radiotherapy based on the Geant4 simulator (Allison *et al* 2016). The preliminary dataset consisted of 4.2 million rows with 6 different distributions describing physical properties of the optical photons (*X* and *Y* coordinates, 3D directions and kinetic energy) after their transport within a specific crystal configuration and detection by the photodetector face, a sensor that converts photon energy to electric signals. The model gave promising results with an average similarity of 93.5%, estimated through the Jensen–Shannon distance (Arjovsky *et al* 2017), among all the simulated and GAN-generated distributions. Moreover, the GAN sped the optical photon distribution generation by up to two orders of magnitude. The final goal is to directly integrate the optiGAN within GATE/Geant4 to skip the optical photon tracking process and speed up the optical Monte Carlo simulations. Numerous applications (large detectors, bright scintillators, Cerenkov-based timing positron emission tomography) can benefit from these improvements (Trigila *et al* 2023).

Nevertheless, it took approximately one month to train the model on a x86_64 Intel(R) Core (TM) i9-10 900X central processing unit (CPU) for 10 000 epochs (4 mins 30 s/epoch) with a batch size of 420 000. Since we need optiGAN to be tested on multiple crystal configurations and applications, it is undesirable to train the model on CPU. CPUs are general-purpose processors that are designed to execute complex tasks like running the operating system (OS), managing memory, executing programs and other latency-sensitive tasks.

Graphics processing units (GPUs) are specialized hardware originally designed with hundreds of small cores called CUDA cores (Luebke 2008), a functional unit that executes instructions for one GPU thread, to handle graphics-intensive tasks. A group of CUDA cores and memory units form a single-instruction-multiple-data (SIMD) unit called a streaming multiprocessor (SM) responsible for executing a CUDA kernel. GPUs have been reconfigured to perform other complex and parallel calculations such as tensor operations, machine learning, and scientific computations due to their high parallel processing capabilities.

Training a deep learning model predominantly involves a lot of large matrix multiplications, which makes it ideal to use GPUs since they excel at performing such calculations. GPUs are also very efficient at processing large data samples at a time since they have a high memory bandwidth (i.e. the number of memory transactions per unit time from/to the memory to the cores). However, it requires proper memory management, efficient data transfers, leveraging model and GPU optimizations to maximize the GPU performance. It is crucial to avoid improper techniques that may worsen the training performance.

In this paper, we discuss and analyse different optimization techniques we used to modify optiGAN original model for GPU, to train it and have significant improvements in the model execution time and memory footprint, while maintaining high fidelity. The objective is to provide a clear understanding of training a model on a GPU and provide a comprehensive performance analysis and evaluation of these techniques to achieve runtime performance improvements. Section 2 provides an overview of the architectural differences between CPU and GPU, a background on the optiGAN architecture, dataset, and some relevant concepts, and the appropriate frameworks and libraries to be used to avoid compatibility issues with the GPU. In section 3, we show the results from all the optimizations and finally discuss about the upcoming GPU technologies and how they can further improve the training performance of deep learning models. Improving the speed of optGAN with GPU can drastically change the use of nuclear imaging system optical simulations by enabling high-fidelity system-level simulations in reasonable training and computation times.

## 2. Materials and methods

### 2.1. CPU vs GPU architecture

**Figure 1.**
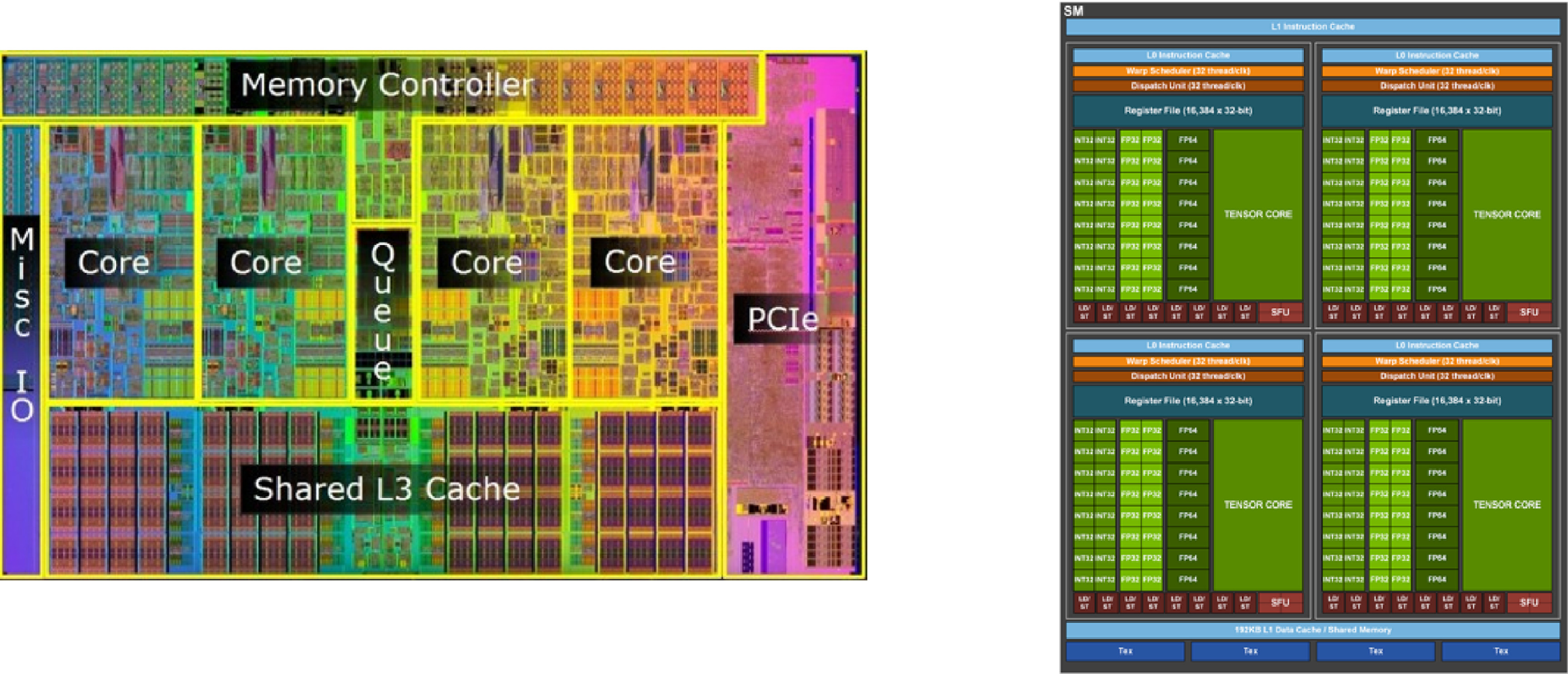
CPU and GPU architectures.

CPUs consists of small number of powerful cores that excels at tasks that require efficient single-threaded performance and handle a wide range of general-purpose computing workloads (figure 1 left). CPUs typically have sophisticated memory hierarchies, including multiple cache levels, that minimizes the memory latency as much as possible and improve data access. Their design prioritizes versatility, which enables them to execute diverse instructions and manage complex tasks efficiently.

In contrast, GPUs are specifically designed to handle highly parallelizable tasks (figure 1 right). They have a larger number of smaller and less powerful cores optimized for simultaneous execution. GPUs are particularly suited for graphics rendering, scientific simulations, and compute-intensive workloads like deep learning that involve extensive parallel computations. Their architecture enables them to process large amounts of data in parallel, leveraging high memory bandwidth to efficiently perform memory operations on the data. While GPUs may have slightly higher memory latency compared to CPUs, they try hiding the latency through multithreading.

These features offered by the GPU are harnessed for deep learning applications by integrating deep learning frameworks with the CUDA API. CUDA (Compute Unified Device Architecture) (Luebke 2008) is a parallel computing platform and programming model developed by NVIDIA. By utilizing CUDA, the deep learning frameworks optimizes the execution of operations such as matrix multiplications and convolutions, which are fundamental to deep learning, resulting in efficient utilization of GPU resources and improved overall performance.

Overall, the architectural differences between CPUs and GPUs are based on their their respective optimization goals. CPUs prioritize versatility and single-threaded performance, making them well-suited for general-purpose computing tasks. GPUs, on the other hand, are designed for parallel processing, making them highly efficient for tasks that can be divided into smaller, independent operations.

### 2.2. ML training on GPU

#### 2.2.1. Dataset

**Figure 2.**
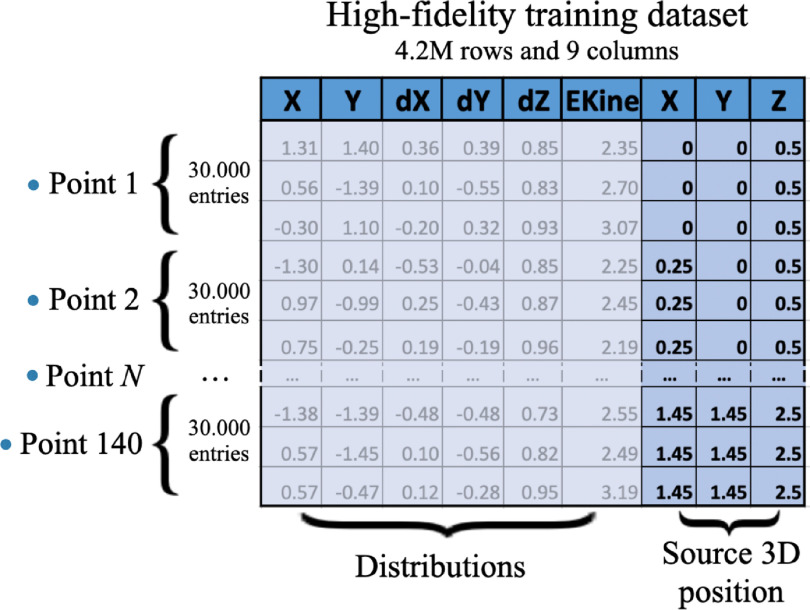
OptiGAN training dataset. Accurate optical simulations were performed at different emission positions inside a crystal to train and test the conditional generative adversarial network optiGAN. Optical photon distributions (position, directions, and energy) were stored for 140 emission points in a multidimensional matrix which included the source 3D emission positions. This tabular data was used as the high-fidelity training dataset of the optiGAN.

The training dataset, figure 2, consisted of 140 simulated emission positions in the crystal volume, representing 1/8 of the crystal cross-section and five depths of interaction. Additionally, 1000 separate optical photons emission points were randomly selected for testing the GAN’s prediction capabilities. Each position generated at least 30 000 optical photons on the photodetector face. The tabular data of these points included the *X* and *Y* coordinates, 3D directions (dX, dY, dZ), kinetic energy (EKine), and a class label indicating the 3D emission source position. This data was concatenated to form a single training matrix with nine columns and 4.2 million rows, allowing us to train the Wasserstein Conditional GAN. More details can be found in Trigila *et al* (2023).

#### 2.2.2. GAN architecture

**Figure 3.**
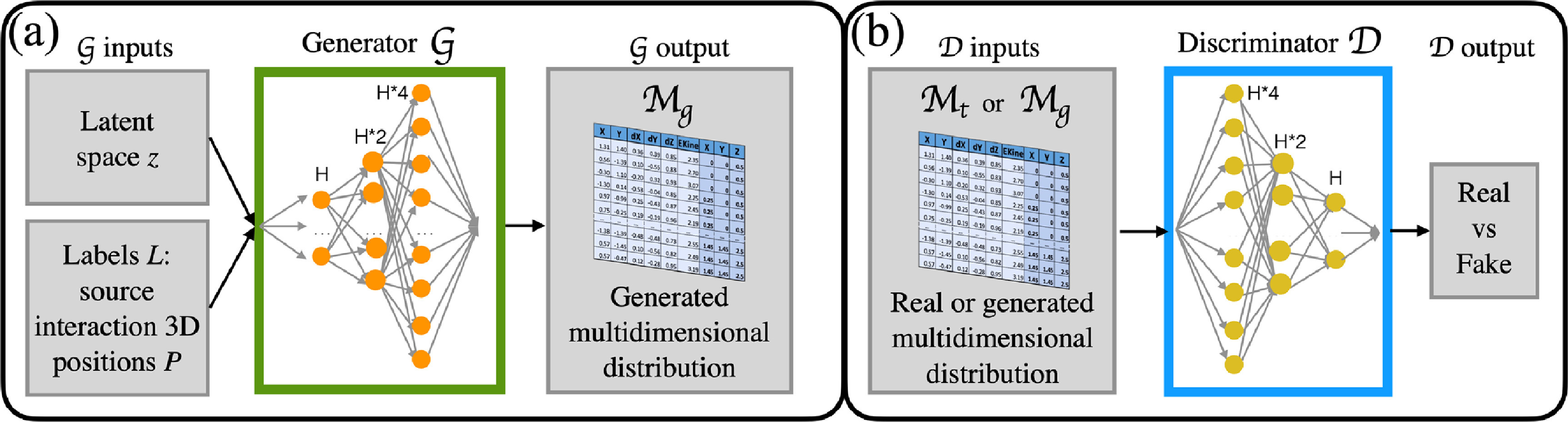
OptiGAN architecture (from Trigila *et al* (2023)). It consists of a Generator (a) and a Discriminator/Critic network (b) with *H* = 128 hidden nodes and a ReLU activation function.

Figure 3 shows the architecture of optiGAN. It comprises two separate neural networks, the Generator (G) (figure 3(a)) and Discriminator/Critic (D) (figure 3(b)). We used the Wasserstein (or Earth Mover’s) distance as loss function (Arjovsky *et al* 2017) together with GP (Gulrajani *et al* 2017), which both were shown to provide better GAN learning stability than the conventional binary cross entropy loss function and gradient clipping (Arjovsky *et al* 2017). Together with improved stability of the optimization process, it was shown that the Wasserstein loss metric correlated with the generator’s convergence and sample quality (Arjovsky *et al* 2017).

#### 2.2.3. Model training

The optiGAN model, after the GPU optimizations that will be described in next paragraph, was trained for 10 000 epochs with a batch size of 250 000 samples. Adam optimizer was used to minimize the loss function and the hyperparameters *β*1 and *β*2 were set to 0.5 and 0.9, respectively. The learning rate was chosen empirically and set to 0.000 05. For every batch, the Critic network was trained thrice and the Generator network was trained once. The training of the optiGAN model was carried on an 8GB Nvidia Quadro RTX 4000 GPU (Data Sheet: Quadro RTX 4000 2019).

When training a deep learning model on GPU, it is essential to ensure that the library versions are compatible to avoid potential conflicts or errors. The compatibility requirements generally involve the versions of GPU driver, PyTorch/Tensorflow, CUDA (Compute Unified Device Architecture), and cuDNN (CUDA Deep Neural Network library) (Chetlur *et al* 2014). Table 1 shows the versions of different frameworks and tools we used to perform the training. It is best to create different virtual environments for different projects according to the specific requirements. This will help avoiding version compatibility issues.

**Table 1.** Frameworks and library versions used to train the GAN on GPU.

GPU Driver	Python	CUDA	cuDNN	PyTorch
470.182.03	3.9.13	11.4	8.3.2	1.13.0

To train the optiGAN model on the GPU the following optimizations changes were made to maximize the GPU performance:

• Multiprocess data loading (4 CPU workers) for efficient data transfers
• Optimizing the batch size based on the execution time and model performance trade-off
• Automatic mixed precision (AMP) to increase the GPU utilization and reduce memory footprint

In the next section, the above optimization methods are explained briefly.

### 2.3. GPU optimization techniques

#### 2.3.1. Dataloader

Dataloader is responsible for feeding batches of the pre-processed dataset into the deep learning model during training. Dataloader optimization can significantly improve the training of a deep learning model on a GPU by enhancing data loading efficiency, memory usage, and overall training performance (Svogor *et al* 2022). We leveraged Pytorch’s inbuilt DataLoader module (Torch.utils.data 2023) to spawn multiple CPU workers to load the data in parallel by tuning the ‘num_workers’ parameter.

To optimize dataloading and training a deep learning model on a GPU, several techniques were used. First, using a multiprocess DataLoader can parallelize data loading, reduce the CPU load, and speed up the process. Second, using data prefetching significantly reduces the data loading duration in between the batches by loading the next batch on CPU while the current batch gets processed on the GPU. Data prefetching is a memory optimization technique used in modern GPUs to enhance memory access performance. In GPUs, memory access latency can be a bottleneck in fully utilizing their processing power. Data prefetching aims to solve this bottleneck by fetching data from the GPU’s cache or memory before it is actually needed for computation. It is particularly important in GPU-intensive workloads like deep learning and AI, where neural networks require frequent access to large datasets. Effective prefetching can optimize training performance (Hijma *et al* 2023).

#### 2.3.2. AMP

**Figure 4.**
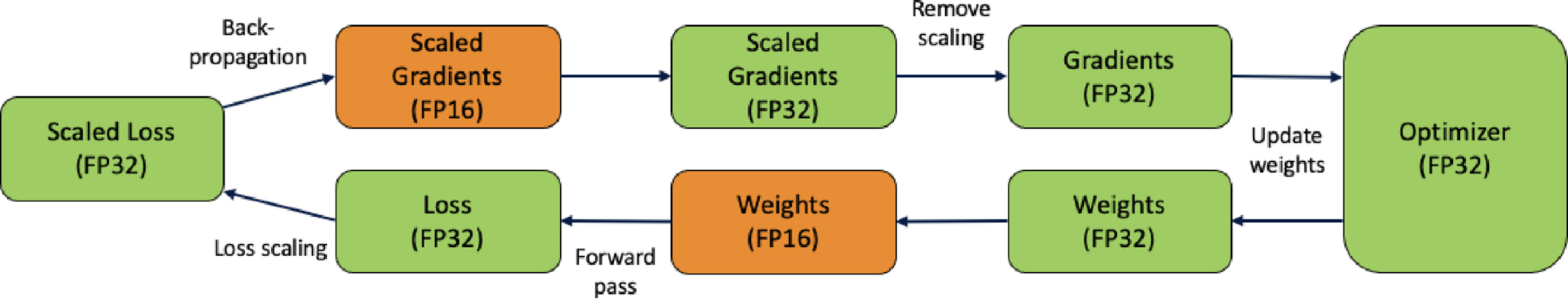
Automatic mixed precision training pipeline.

AMP (Micikevicius *et al* 2018) is a technique that intelligently manages the precision of computations during deep learning model training. It does this by using two different levels of precision in calculations: a higher one called single-precision (FP32) and a lower one called half-precision (FP16). The Weights, activations and gradients of the model are stored using FP16, and an FP32 master copy of weights is used for updates. This helps speeding up the training while keeping the numbers stable. AMP efficiently scales the precision of model parameters and activations by leveraging the Tensor Cores in the GPU. To prevent issues like gradient underflow and overflow, AMP uses loss-scaling, i.e. multiplying the loss function by a constant factor. Figure 4 shows the pipeline of the AMP technique. By automatically adjusting precision, AMP significantly speeds up training, reduces memory consumption, and allows for higher batch sizes, making it a powerful method for optimizing deep learning workflows on modern GPUs (Micikevicius *et al* 2018).

#### 2.3.3. Batch size

Batch size is one of the main hyperparameters that will directly affect the GPU memory usage and computation time. Choosing a very high batch size will result in poor generalization of the model and on the contrary, choosing a very low batch size might not effectively use the compute power of the GPU. It is important to find the best batch size that gives the fastest execution time without much compromise in the performance of the model. The optimal batch size depends on various factors, including model complexity, dataset size, and available hardware. It is recommended to experiment with different batch sizes and observe the effects on training performance to determine the best batch size for specific deep learning task on the GPU. For Batch Size optimization, we trained the optiGAN model with different batch sizes to find the optimal one that maximizes the GPU utilization without affecting the convergence of the model.

We used the Nvidia Nsight Profiler tool (Nsight Systems 2018) to understand the areas that required optimization to enhance the performance of the workload and Nvidia Nsight Compute (Nsight Compute 2023) to understand the performance of the major kernels. To annotate specific events in the timeline like Data Loading, Discriminator Iter, Generator Iter, Batch, and Epoch we used the NVTX API (NVTX NVIDIA Tools Extension Library) in Python. This was useful in better visualizing the characteristics of the events. The main GPU traces we used to analyze were:

• Memory usage, this is the GPU memory used by the process through device variables and CUDA APIs
• SM active, the total number of cycles the SMs, Streaming Multiprocessors, are active
• SM instructions, the rate at which the instructions are being executed per cycle
• Tensor Active/FP16 Active, the number of cycles the tensor cores are active during the event
• Compute throughput %, the rate at which the GPU performs arithmetic operations. It is measured by the ratio of FLOPs (Floating Point Operations per second) and the theoretical peak FLOPs of the GPU
• Memory throughput %, ratio of the data transferred between the GPU memory hierarchy and the peak data transfer in the GPU.

## 3. Results

In this section, first we show the execution time results before the GPU optimization. We explain the drawbacks of CPU and an inefficient model training on GPU when not optimized. This will give an understanding as to why training a deep learning model on a GPU with proper optimization techniques is important. Second, we analyze and discuss the results of the GPU optimization techniques used to train the optiGAN model. The results of the different optimization techniques are discussed and how each one will help in mitigating the issues.

### 3.1. CPU vs GPU

Table 2 shows the execution time per epoch, the time taken to process the whole dataset to train the model, on CPU and GPU using Keras and Pytorch libraries before applying the GPU optimizations. The execution time is high when the model is trained on both CPU and GPU using the Keras library, more than 9 and 6 min respectively. There was a significant decrease in the execution time when the model was trained on CPU and GPU in Pytorch, 3 min and 30 s and 43 s, respectively. Hence, we decided to migrate to Pytorch which is a low-level API to train deep learning models. Additionally Pytorch offers more flexibility and control over training and it is relatively easier to code when compared to Tensorflow. Using Pytorch, there was approximately a 5x improvement in the execution time when training the model on GPU as opposed to CPU. Nevertheless, it took 5 days to train the model for 10 000 epochs on GPU. We investigated how the GPU optimizations could further decrease the execution time. Hence, we first analyzed the main GPU bottlenecks using the Nvidia Nsight profiling tool and then used the respective optimization techniques to reduce the bottlenecks.

**Table 2.** Execution time results (Batch size-100 000) before the GPU optimization.

	Keras (CPU)	Keras (GPU)	Pytorch (CPU)	Pytorch (GPU)
Execution Time/Epoch	9 mins 22 s	6 mins 30 s	3 mins 30 s	43 secs

### 3.2. Nsight systems profiler results

**Figure 5.**
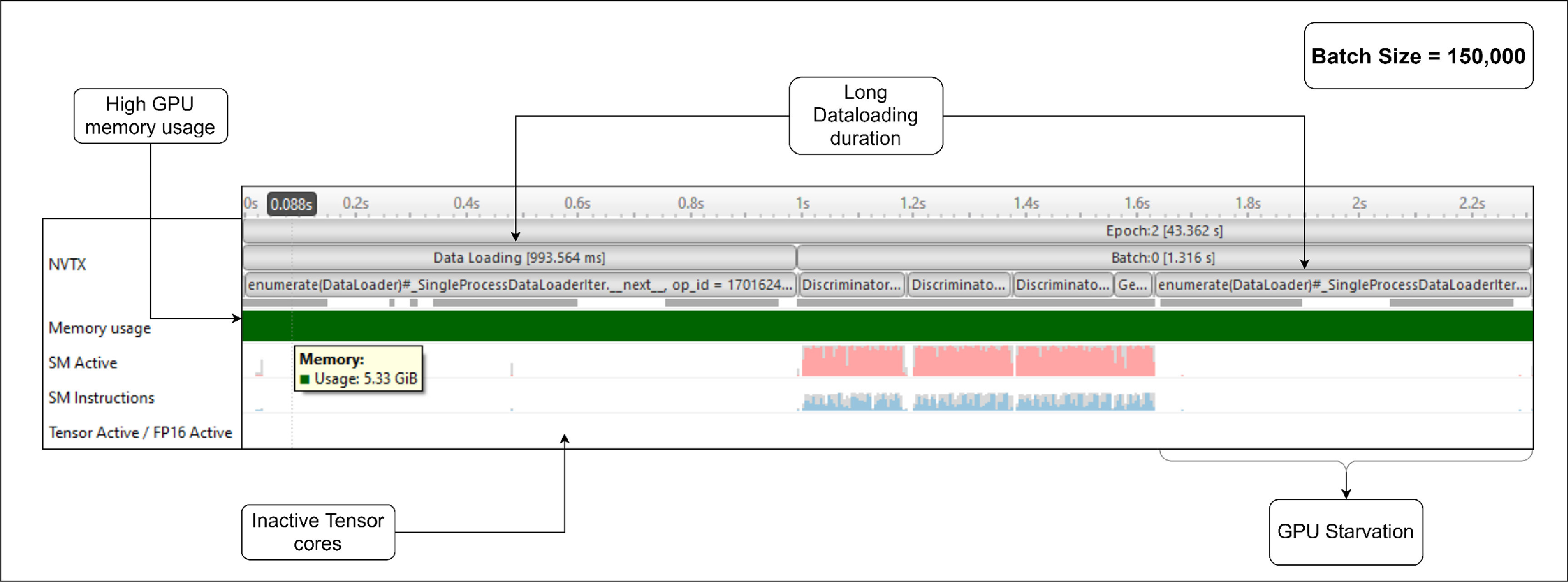
GPU Profiling results using Nvidia Nsight Systems. It shows the runtime performance of different sections of the model training (like Dataloading, Generator and Discriminator training process), GPU memory usage, GPU utilization (SM active and SM instructions), and the tensor core activity. These events were sampled at a rate of 10 kHz. The pink regions consists of several vertical lines which represents the percentage of the GPU cores that were active at that moment and the the blue regions represents the percentage of instructions issued by the SMs at that moment.

Figure 5 shows a snippet of the first dataloader batch and batch processing of the model training on the GPU with a batch size of 150 000 (value used before the GPU optimization). On average, it took around 43 s to process one epoch. In the initial data Loading phase, which lasted 993 ms, the dataset gets split into batches and the dataloader loads the first batch. Then, for the subsequent batches, 1.3 s were spent to process single batch of data. More than half of this time was spent on loading data of the next batch (700 ms). This is due to single process dataloader used for loading the whole dataset. This also results in the GPU being idle for more than half of the training time. This is called GPU starvation. The GPU memory usage (5.53 GiB) is also high for a batch size of 150 000. This was the maximum batch size that we could set before the GPU ran into Out-Of-Memory.

It is also seen that the tensor cores of the GPU were inactive throughout the training. Using the tensor cores would improve the memory usage and the processing time of the training since they are specially designed to execute multiple operations in one clock cycle. Hence, they are the best to handle deep learning computation workloads.

To reduce the effects of these problems we explored the three different GPU optimization techniques presented in section 2.3.

### 3.3. GPU optimizations results

#### 3.3.1. Dataloader

**Figure 6.**
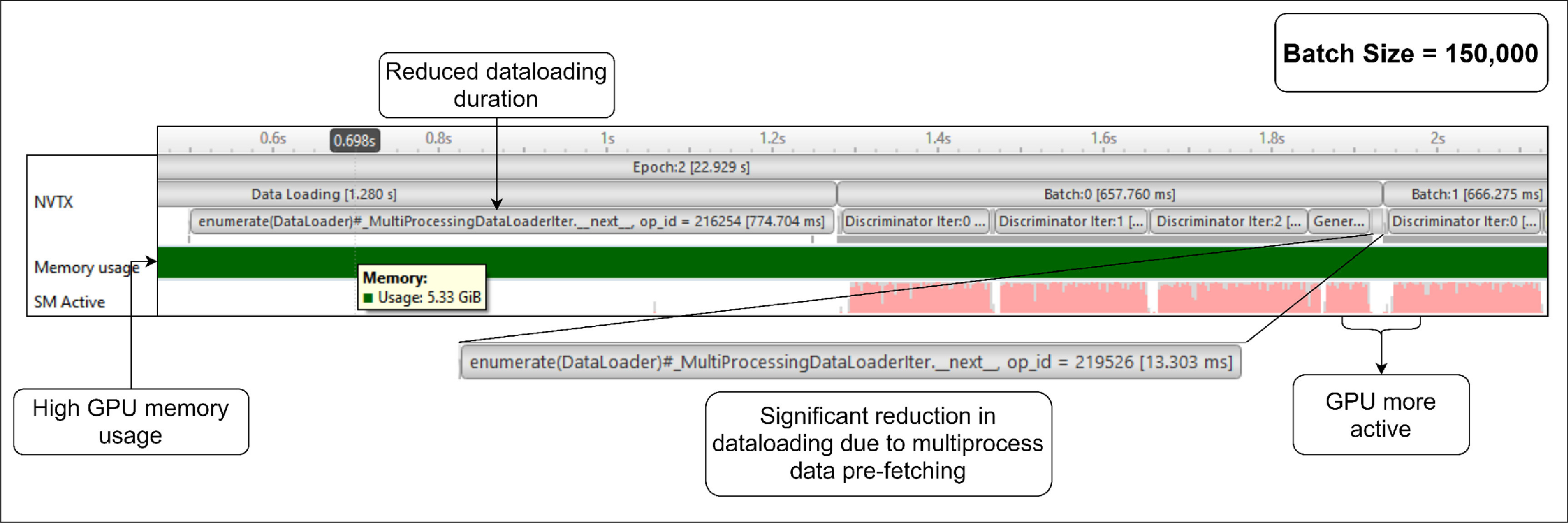
Dataloader optimization results. The runtime (22.9 (s)) dropped by almost 2 times from the previous GPU runtime showed in figure 5. The GPU is active during most of the training duration. However, the memory usage was not optimized with this technique.

Optimizing the dataloader solved the problem of GPU starvation and reduced the data loading phase duration in between the batch computations. By enabling multiple CPU workers (4 CPU workers) to load the dataloader, the initial phase took around 1.28 s and using only 774 ms to load the batch and the rest of the time could have been spent on initializing and spawning the CPU workers. There was a significant decrease in average loading time of the subsequent batches, from 700 ms to 15 ms, this reduced the average computation time per batch to 650 ms. As a result of this, the GPU was kept busy most of the time. Consequently, the execution time per epoch was reduced down to 23 s compared to the previous 43 s as shown in (figure 6).

#### 3.3.2. AMP

**Figure 7.**
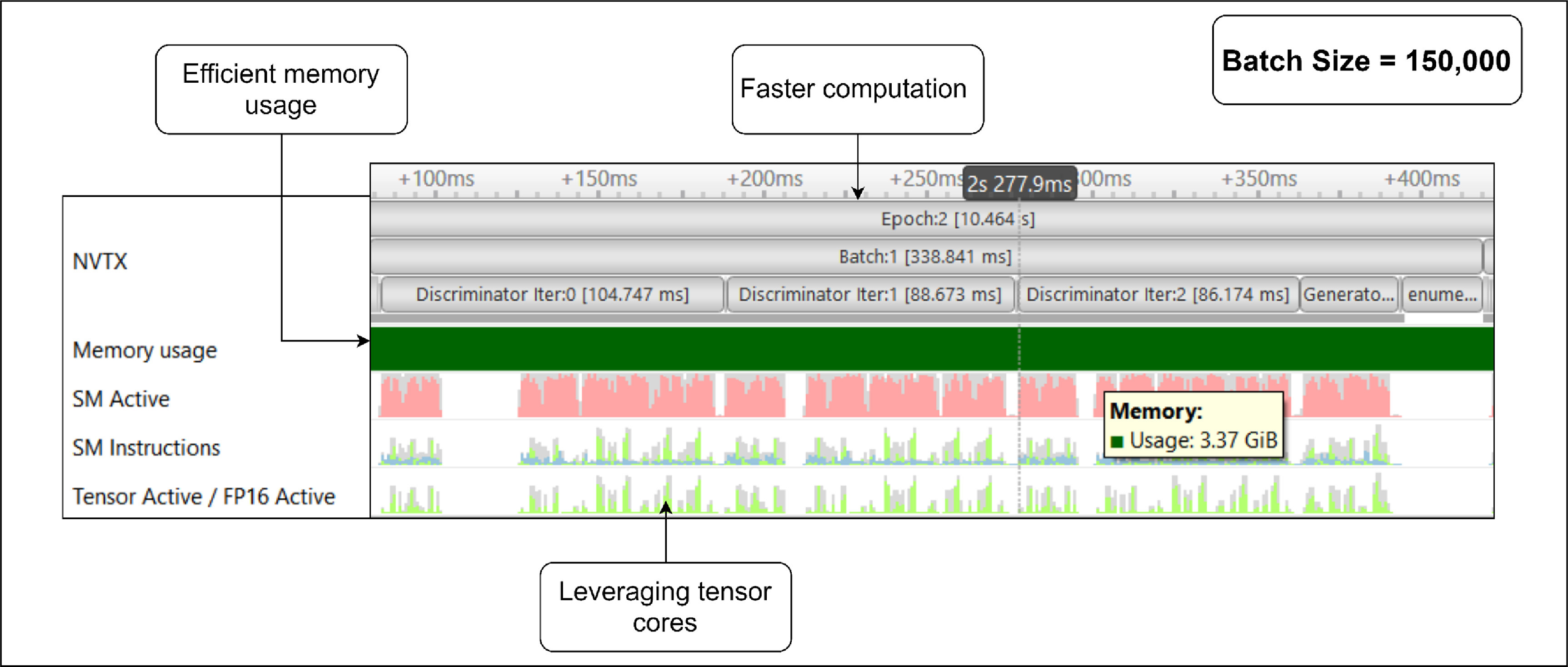
Automatic Mixed Precision optimization results. The runtime was further reduced (10.4 (s) and the memory usage was reduced to 3.37 GiB using tensor cores, that specializes in optimizing deep learning computation. It also gives the possibility to increase the batch size.

Figure 7 shows the profiler snippet of the model training after using the AMP technique (along with the Dataloading optimization). This successively resulted in a lesser time taken to process one batch of data, 339 ms, due to the reduced precision of the model and activation functions. The GPU memory footprint was reduced (3.37 Gib), which allows the batch size to be increased further than 150 000. The tensor cores were also used in computing the intermediate matrix multiplications hence reducing the computation time of the batches. The execution time was bought down to 10.464 s. Additionally, there was an increase in the model performance, to an average Jensen–Shannon similarity value of 94.5%.

#### 3.3.3. Batch size

The next goal was to choose the optimal batch size to train the model.

**Figure 8.**
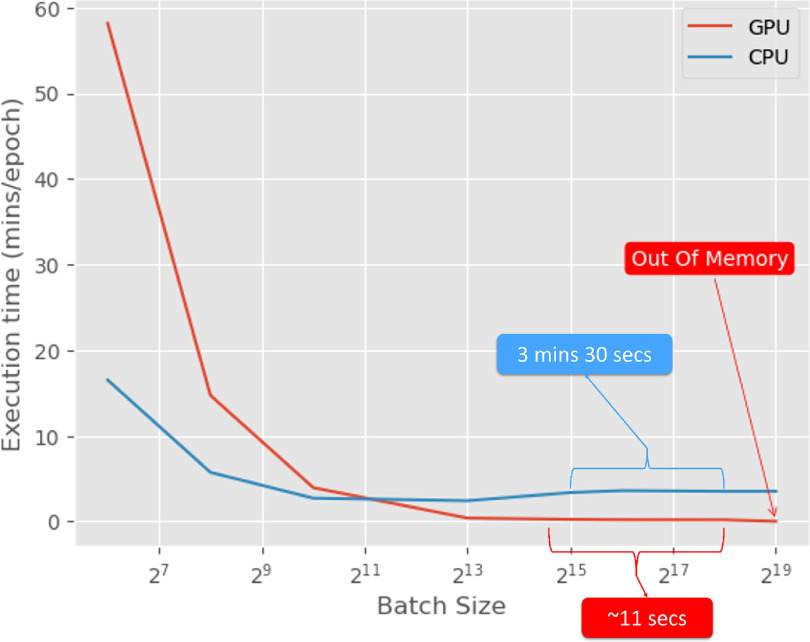
optiGAN model execution time and batch size comparison in CPU and GPU.

We plotted an execution time per epoch as a function of the batch size graph on both CPU and GPU (with Dataloader and AMP optimization) (figure 8). On the CPU, with an increase in the batch size, initially there was a steady drop in the execution time due to parallel processing in the cores. Around batch size of 2^10^, the curve started plateauing with an execution time around 2 mins 24 s. This indicates that maximum parallelism on the CPU can be achieved only between a batch size of 2^10^ and 2^13^. In the end there is a slight increase in the execution time which could be due to pipelining of the parallel execution since it already reached its maximum capability. The GPU curve showed an exponentially decreasing execution time with increasing batch size until the GPU ran out of memory. Initially the GPU performs worse than CPU because if the batch size is low, it cannot maximize the parallel processing ability of the GPU. There will also be a Host-to-Device (CPU to GPU) memory transfer overhead due to numerous batch transfers to the GPU. After a batch size of 2^13^, the decrease in execution time starts plateauing and the optimal batch size was found to be between 2^15^ and 2^19^. The optiGAN model was trained with 250 000 and the model performance, evaluated in terms of similarity by the Jensen–Shannon distance, remained unaffected around the value 94.5%.

#### 3.3.4. Other optimizations

We tried using other optimization techniques, like Batch Accumulation and Pinned Memory, which did not give any improvement in the model performance. However, we replaced the traditional Adam optimizer with the Fused Adam optimizer to try speeding up the optimizer step function execution. The fused Adam optimizer is designed to accelerate the training by combining multiple computations in a single step. There was a decrease in the execution time from 3.456 ms to 1.262 ms, which did not make much difference to the overall training time. This optimization will be useful when working with large models and datasets, as it can significantly speed up training times and reduce memory consumption. However, it is important to use this carefully since it could affect the performance of the model.

#### 3.3.5. Kernel execution summary

The Nsight System profiler tool provides the kernel trace information, which sequentially lists the executed kernels during the model training, the number of times the kernels were invoked and the average execution time for each kernel. This information can be used to get an idea of the most-used kernels and further analyse the performance of the individual kernels using the Nsight Compute tool.

Table 3 shows the kernels that were executed majority of the optiGAN training (with AMP, Batch size optimization and Multiprocess dataloader) duration. The most frequently executed kernel was the vectorized elementwise kernel. From the Compute throughput % and the Memory throughput % metrics, it is clear that this kernel is Memory bound, i.e. there were more data transfer operations than arithmetic operations during this kernel execution. On the contrary, the turing fp16 and turing fp16 gemm kernels are compute bounded, i.e. more arithmetic operations were executed than data transfers.

**Table 3.** Major kernels executed.

Time	# of kernel instances	Compute throughput (%)	Memory throughput (%)	Kernel name
16.3%	867	24.63	90.35	vectorized elementwise
7.6%	340	47.46	83.22	fused dropout
7.0%	238	61.00	42.70	turing fp16 gemm
4.7%	170	66.85	43.35	turing fp16 gemm relu

Ideally, to get the best runtime time performance, majority of the kernels would have to be compute bounded. But in reality, the GPU has limited memory bandwidth and will eventually take some time to move around the data. Predominantly, the GEMM (General Matrix Multiplication) kernels are executed in the tensor cores in Quadro RTX 4000 GPU, but in this case the GEMM kernels are not utilizing the most out of it. This indicates that the GPU is capable of process more data and increasing the batch size or increasing the model size, if required, will improve the GPU performance. Similarly, the total number of vectorized elementwise kernels executed can be reduced by reducing the number of memory operations as much as possible.

## 4. Discussion and conclusion

**Figure 9.**
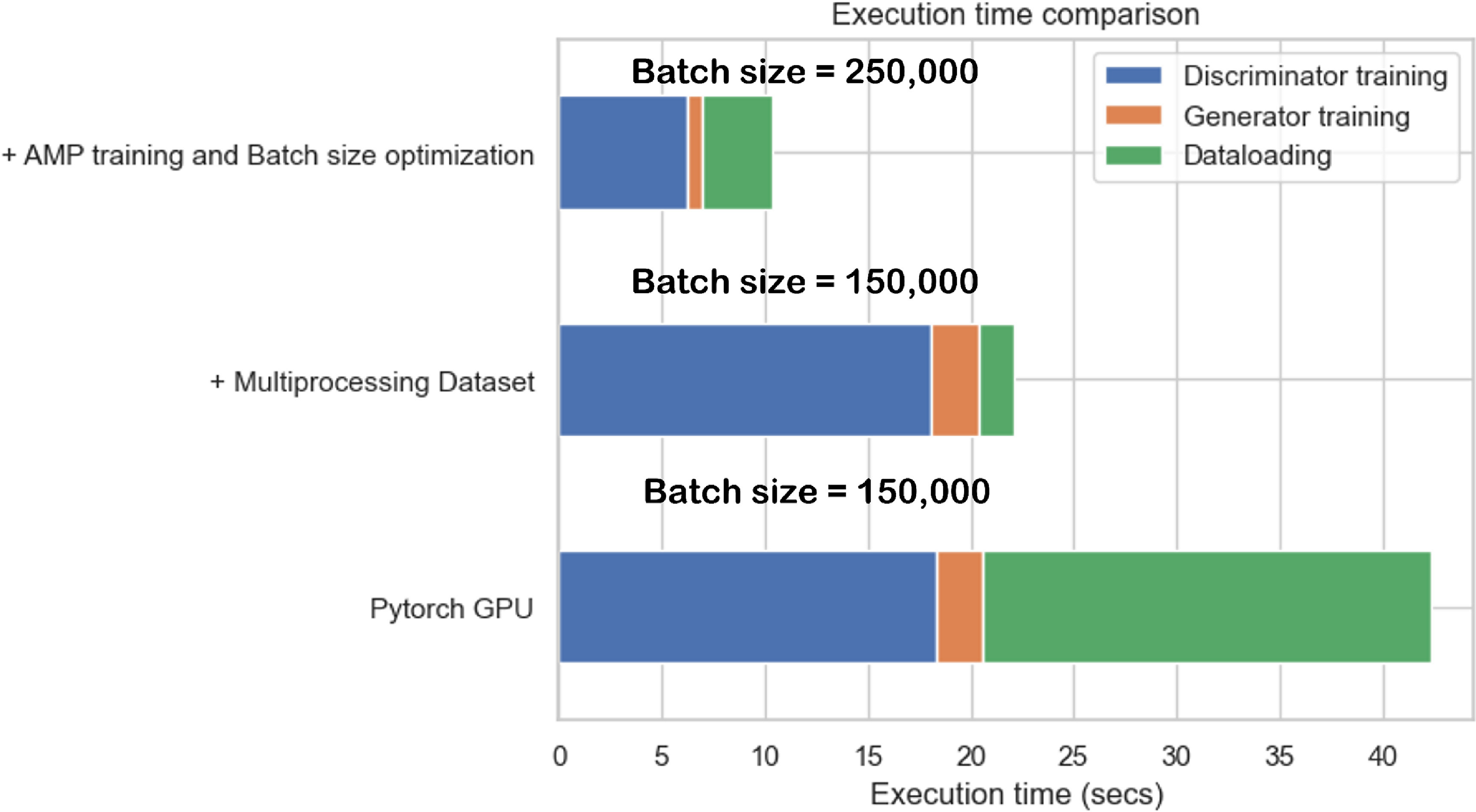
Execution time comparison of the GPU optimizations.

Using some effective GPU optimization techniques we were able to accelerate the training of the optiGAN (Trigila *et al* 2023) model without any loss in the model’s performance measured by the Jensen–Shannon similarity value. Figure 9 shows the comparison plot of all the techniques with different runtime regions during the training. As expected, after the dataloader optimization, the dataloading duration got reduced by a large margin. Using the AMP and batch size optimizations, we were able to achieve further reduction in the processing duration of the Generator and Discriminator networks. The execution time was reduced to 10 s which is almost 4.5 times that of an inefficient model training and almost 20 times that of the model trained on a CPU.

Although customized to a new application in the field of medical physics, optiGAN employs a combination of established structures, and the optimization techniques and ML workload performance metrics used in this study are applicable across different neural network architectures and applications.

While we achieved a significant improvement in the overall execution time, there are some limitations of the used optimization techniques and the profiling tools. The AMP training gave the most performance improvement for execution time, GPU memory and GPU starvation time. The major drawback of using AMP training was that it was prone to numerical instability due to reduced precision. This was tackled by using a gradient scaler function to minimize under flowing of gradients. Similarly, while training the model with large batch sizes, it was important to choose a batch size such that the GPU utilization was maximized with minimal loss in the model performance. Apart from this, the latency caused due to Nsight Systems and Nsight Compute profilers might disturb the actual workload.

In the next phase of the research, we aim to scale the GAN model to train on multiple crystal configurations and test the capability of the model to learn features of different crystal types and configurations. To get the best performance from the model, we are planning to leverage Hyperparameter Tuning algorithms. Further techniques will be explored to increase the usage of the tensor cores, reduce the data transfer between CPU and GPU, reduce the memory footprint of the model, and improve the Compute and Memory throughput. We are also seeking to enhance the accuracy and robustness of the generated probability distribution by stacking multiple deep learning models into an ensemble. This would require exploiting model and data parallelism, using distributed training strategies (Mittal and Vaishay 2019) and multiple GPUs to train the model.

With increasing interest in Generative AI, specifically LLMs (Large Language Models), and rapidly increasing model size, the demand for AI Hardware is exponentially increasing (Kaplan *et al* 2020) for both training and inference. This increased demand is one of the major contributing factors to the current GPU shortage and undesirable carbon emissions (Patterson *et al* 2021). Companies like Nvidia and AMD have been focusing on tackling these concerns to specifically tailor GPUs (like Nvidia A100, H100, AMD MI200, MI300) to efficiently handle AI workloads with higher compute capacity and power efficiency.

While increasing the processing power of the hardware is important, developing efficient software to execute on the hardware is equally important in reducing the computational workload and memory usage. Hence, writing efficient code, leveraging optimized libraries, and exploring algorithmic improvements to harness the full potential of GPUs, can lead to substantial gains in performance and productivity.

Training of large models in lesser precision formats like FP16 (the one used in this work), BF16 (Brain Float-16), INT8 (Integer-8), and even 1-bit training (Ma *et al* 2024) has increased and gives major performance improvements with the memory throughput, compute utilization and decreased size of the model. FP16 was used in this work and we did not require using lower precisions to maintain the generation capability of the model. Similar to data prefetching on the CPU using multiple workers, the utilization of memory prefetching (Van der Wijngaart and Oh 2022) in GPU has been found to improve the computation speed of the GPU kernels by hiding the memory latency. These state-of-the-art techniques will be explored in future studies related to fine-tuning and scaling the optiGAN model to extend its generation capability and across different architectures to validate the generalizability of these techniques.

In other works related to the optimization techniques used in this paper, but not applied to GAN, authors have proposed an improved version of the Pytorch’s dataloader performance using (Svogor *et al* 2022). They have managed to reduce the batch loading time upto 12x when compared to the current implementation of the Pytorch DataLoader. Next steps of our work will include testing this new dataloader, although we have already achieved very good results with the original version of Pytorch’s dataloader.

Other techniques exist and are in rich development nowadays (Flash Attention-2 (Dao 2023), Sparse Computation optimization (Kundu *et al* 2023)), but they did not apply to our model since they are predominantly used to large language models which are larger and more complex models than optiGAN.

Last, the final goal is to integrate the model into the simulation toolkit GATE, which is an open-source toolkit hosted on GitHub. When optiGAN is integrated in GATE, and thoroughly tested, it will be freely accessible on GitHub (source code, training dataset, trained model).

## Data Availability

The data cannot be made publicly available upon publication because they are not available in a format that is sufficiently accessible or reusable by other researchers. The data that support the findings of this study are available upon reasonable request from the authors.
